# Nanoencapsulation of *Helichrysum italicum* (Roth) G. Don essential oil for eco-friendly control of citrus aphid (*Aphis spiraecola*)

**DOI:** 10.3389/fpls.2026.1808571

**Published:** 2026-04-13

**Authors:** El Khansa Bourenane, Dalila Amokrane, Dikra Bouras, Xiankun Wu, Ahmed Mohammedi, Mamoun Fellah, Christian Andreasen

**Affiliations:** 1 Department of Agronomy, Laboratory of Natural and Local Bio-resources, Hassiba Ben Bouali University, Chlef, Algeria; 2 Department of Agronomy, Laboratory of Plant Production and Protection of the Chlef Region, Hassiba Ben Bouali University, Chlef, Algeria; 3 Department of Science of Matter, Faculty of Science and Technology, University of Souk-Ahras, Souk Ahras, Algeria; 4 Department of Physics, Chemistry and Pharmacy, University of Southern Denmark, Odense, Denmark; 5 Department of Mechanical Engineering, University of Abbes Laghrour, Khenchela, Algeria; 6 Department of Plant and Environmental Sciences, University of Copenhagen, Copenhagen, Denmark

**Keywords:** botanical insecticide, chitosan nanoparticles, citrus plant protection, citrus production, nanotechnology

## Abstract

**Introduction:**

Sustainable citrus production requires effective alternatives to synthetic insecticides. *Helichrysum italicum* essential oil (EO) has demonstrated insecticidal potential, but its volatility and rapid degradation limit practical application. Nanoencapsulation offers a strategy to enhance EO stability and efficacy.

**Methods:**

EO was nanoencapsulated in chitosan nanoparticles using ionotropic gelation. Physicochemical characterization assessed encapsulation efficiency, colloidal stability, and particle size distribution. *In vitro* bioassays against *Aphis spiraecola* compared nanoencapsulated EO with emulsified EO. Semi-field trials evaluated efficacy under environmental stress. Molecular docking was performed to explore interactions between EO constituents and acetylcholinesterase.

**Results:**

Nanoencapsulation achieved high encapsulation efficiency (97.9%), strong colloidal stability (+45.5 mV), and uniform particle size (173.4 nm; PDI 0.27). *In vitro* assays revealed concentration- and time-dependent aphid mortality, with nanoencapsulated EO significantly more potent (LC_50_: 0.48 mg/mL at 90 h; 0.35 mg/mL at 96 h) than emulsified EO (LD_50_: 8.26 μL/mL at 60 h). Semi-field trials confirmed dose- and time-dependent mortality, though efficacy decreased by 10–28% under UV and oxidative stress. Molecular docking indicated EO constituents (caryophyllene, linalool) interact with acetylcholinesterase, supporting a neurotoxic mode of action.

**Discussion:**

Nanoencapsulation improved EO stability, photoprotection, and residual activity, enhancing its insecticidal performance compared to conventional formulations. Despite reduced efficacy under environmental stress, the formulation demonstrated promising potential as a plant-based pesticide. These findings support the integration of nanoencapsulated EO into sustainable citrus pest management strategies.

## 1 Introduction

Citrus production is a major sector of global agriculture, yet its sustainability is increasingly threatened by insect pests (Fernández-Echeverría et al., 2024). The widespread use of synthetic pesticides has historically boosted crop yields, but their overreliance has generated severe ecological and agronomic consequences, ultimately jeopardizing long-term agricultural sustainability (Quandahor et al., 2024; Tudi et al., 2021). Carbamates, pyrethroids, and neonicotinoids remain dominant in pest management due to their rapid knockdown effects on phytophagous insects (Aioub et al., 2024; Quandahor et al., 2024). However, repeated applications of these chemicals have triggered the evolution of resistant pest populations, disrupted ecosystems by harming beneficial organisms, and caused pervasive environmental contamination through bioaccumulation and leaching into water systems (Quandahor et al., 2024; Tudi et al., 2021). These unintended consequences have intensified the search for sustainable alternatives, particularly botanical pesticides, which offer targeted pest suppression with minimal ecological disruption (Aioub et al., 2024; Assalin et al., 2025; Bourenane et al., 2025).

Among natural pest control agents, plant-derived essential oils (EOs) have gained considerable attention due to their complex chemical compositions, which enable multiple modes of action against insects, reducing the likelihood of resistance development (Aioub et al., 2024; Ebadollahi et al., 2022; Heydari et al., 2020).

The Asteraceae family, comprising over 6000 species, is particularly rich in bioactive compounds such as alkaloids, terpenoids, and flavonoids, which exhibit insecticidal, repellent, and growth-inhibitory properties (Borgo et al., 2024; Bourenane et al., 2025). Within this family, the genus *Helichrysum* stands out for its diverse secondary metabolites, primarily phenolic compounds, which contribute to its antimicrobial, antioxidant, and insecticidal activities (Göre and Avcı, 2022). *Helichrysum italicum* (Roth) G. Don, a Mediterranean aromatic shrub, has been extensively studied for its antimicrobial and antioxidant properties (Węglarz et al., 2022), yet its potential as a botanical insecticide remains underexplored (Furlan and Bren, 2023; Judzentiene et al., 2022). Nevertheless, oxygenated monoterpenes present in its essential oil (Judzentiene et al., 2022) are known to disrupt insect nervous systems through mechanisms including acetylcholinesterase inhibition, GABA receptor modulation, and octopamine receptor activation (Abdelgaleil et al., 2024; Qasim et al., 2024). This suggests that, although the insecticidal applications of *H. italicum* are not fully validated, its chemical profile provides a strong rationale for further investigation. Despite their efficacy, the practical application of EOs is limited by their high volatility, low water solubility, and susceptibility to environmental degradation (Singh and Pulikkal, 2022). To address these challenges, nanoencapsulation techniques have been developed to strengthen EO resilience, bioavailability, and ensure controlled release (Bourenane et al., 2026).

Among biopolymers used for encapsulation, chitosan—a deacetylated derivative of chitin—has emerged as a leading candidate due to its biocompatibility, biodegradability, and functional versatility (Abd El-Monaem et al., 2022; Van Bavel et al., 2023). Its positively charged amino groups facilitate electrostatic interactions with anionic crosslinkers such as sodium tripolyphosphate (TPP), enabling the formation of stable nanoparticles via ionotropic gelation (Marques Gonçalves et al., 2023; Van Bavel et al., 2023). Nanoencapsulation not only protects volatile EO components from degradation but also improves their adhesion to insect cuticles and prolongs their release, improving pesticidal efficacy while reducing application frequency (Aioub et al., 2024).

The need for alternative pest control strategies is particularly urgent for aphids, which rank among the most destructive agricultural pests due to their rapid reproduction and role as vectors for plant viruses (Bhat and Rao, 2020; Farhan et al., 2024). *Aphis* sp*iraecola* (Patch, 1914), the green citrus aphid, poses a severe threat to Mediterranean citrus orchards, where it transmits *Citrus tristeza virus* (CTV), a pathogen responsible for extensive orchard decline and economic losses (Mathioudakis et al., 2025). Conventional insecticides have proven inadequate in managing *A.* sp*iraecola* due to resistance development and non-target effects on natural predators, necessitating the integration of botanical insecticides into pest management programs (Paiva et al., 2024). However, the insecticidal potential of *H. italicum* EO against a key agricultural pest like *A.* sp*iraecola* remains unvalidated. Moreover, the efficacy and mechanisms of a chitosan-nanoencapsulated formulation of this EO have not been investigated, representing a significant gap in translating botanical discoveries into practical, stable agronomic solutions.

Therefore, this study aimed to (i) chemically characterize *H. italicum* EO via GC–MS; (ii) develop and physicochemically characterize an optimized chitosan nanoparticle (CS–NP) encapsulation system for the EO; (iii) evaluate and compare the contact toxicity of emulsified essential oil (EO–Em) and nanoencapsulated EO (CS–EO–NPs) against *A.* sp*iraecola* under controlled laboratory conditions, and assess the efficacy of EO *in vivo* on *Vicia faba* L. plants under semi-controlled conditions; and (iv) employ *in silico* molecular docking to explore the potential neurotoxic mechanism of major EO constituents by analyzing their interactions with aphid acetylcholinesterase (AChE). We hypothesize that nanoencapsulation will significantly enhance the insecticidal efficacy and residual activity of *H. italicum* EO by improving its stability and delivery, as demonstrated *in vivo* assays. The findings aim to provide a scientific foundation for a novel, biorational nanoformulation suitable for integrated pest management programs in citrus cultivation.

## 2 Materials and methods

### 2.1 Chemicals

Chitosan (poly-D-glucosamine, CS) of medium molecular weight was dissolved in distilled water containing 1% (v/v) glacial acetic acid. Sodium tripolyphosphate (Na_5_P_3_O_10_) was employed as the ionic crosslinker, while polysorbate 80 (Tween 80) served as a non-ionic surfactant to enhance formulation stability. Sucrose (C_12_H_22_O_11_) was incorporated as a cryoprotectant to preserve nanoparticle integrity during lyophilization. All chemicals and reagents were of analytical grade and obtained from the Laboratories of Chemistry and Natural Life Sciences, University of Ouled Fares (Chlef, Algeria).

### 2.2 Plant materials

Fresh aerial parts of *Helichrysum italicum* (Roth.) G. Don var. *numidicum* Pomel (Asteraceae) were collected in spring 2025 from the Blidean Atlas region of Algeria (36°15′00″–36°45′00″ N, 2°30′00″–3°50′00″ E). Taxonomic identification was based on morphological characteristics and confirmed by a local botanist, using descriptions from the Algerian Native Plants database and comparison with reference specimens archived in the Chrea National Park herbarium and the herbarium of the Higher National School of Agronomy (ENSA, Algiers, Algeria). No voucher specimen was deposited.

### 2.3 *Aphis* sp*iraecola*

Populations of the green citrus aphid (*Aphis* sp*iraecola* Patch, 1914) were used for bioassays. Specimens were collected in April 2025 from naturally infested citrus leaves in private orchards located in the Mitidja plain (36°45.082′ N, 2°55.296′ E), where aphids represent a persistent pest problem. Field-collected individuals were transported to the laboratory in ventilated containers lined with moistened filter paper to preserve leaf turgor and ensure insect viability. Species identification was performed using the comprehensive taxonomic keys (Blackman and Eastop, 2000), with particular attention to diagnostic morphological features including cauda shape, siphunculi morphology, and antennal tubercle development.

### 2.4 Extraction and chemical analysis of the EO of *H. italicum*

Fresh *H. italicum* leaves were collected and air-dried at room temperature (22 ± 2°C) to preserve volatile compounds before being finely ground using an electric grinder. Essential oil extraction was performed by hydrodistillation in a Clevenger-type apparatus, where 100 g of dried plant material was combined with 1200 mL of distilled water and heated for three hours (Lainez-Cerón et al., 2021). The resulting essential oils were separated from the hydrosol, dried over anhydrous sodium sulfate, and stored in amber vials at 4°C to prevent degradation. Chemical characterization was conducted using gas chromatography–mass spectrometry (GC–MS) at the University of Southern Denmark, samples (0.2 µL) were injected in split mode (0.2 µL at 250°C) onto an HP-5MS column (30 m × 0.25 mm × 0.25 µm) under a constant helium flow (1 mL/min, 99.99% purity). The temperature program began at 50°C (2 min hold), ramped at 3 °C/min to 240°C (10 min hold), and completed after 113 min total runtime. Compound identification was based on retention time (RT) values, molecular weight determination, and spectral matching against the NIST and Wiley databases. Electron ionization mass spectra (70 eV, m/z 35–500) were used to support identification. Relative abundances were calculated by peak area normalization without internal standards, and major constituents (>1%) were confirmed through fragmentation pattern analysis and comparison with published mass spectral data.

### 2.5 Synthesis

#### 2.5.1 Preparation and optimization of chitosan nanoparticles (ionotropic gelation)

Chitosan nanoparticles were synthesized using the ionotropic gelation method, based on electrostatic interactions between chitosan and sodium tripolyphosphate (TPP) (Gutiérrez-Ruíz et al., 2024; Maurizii et al., 2024). Chitosan (0.4% w/v) was dissolved in 1% acetic acid (100 mL) under magnetic stirring overnight. Tween 80 (1% v/v) was added as stabilizer before gradual incorporation of *H. italicum* essential oil (1.5% v/v) under continuous stirring. The crosslinking reaction was initiated by dropwise addition of TPP (0.3% w/v) to the CS–EO mixture while maintaining agitation at 1500 rpm for 60 min. The pH was adjusted to 5–6 using 0.1M NaOH (Gutiérrez-Ruíz et al., 2024).

**Figure 1:**
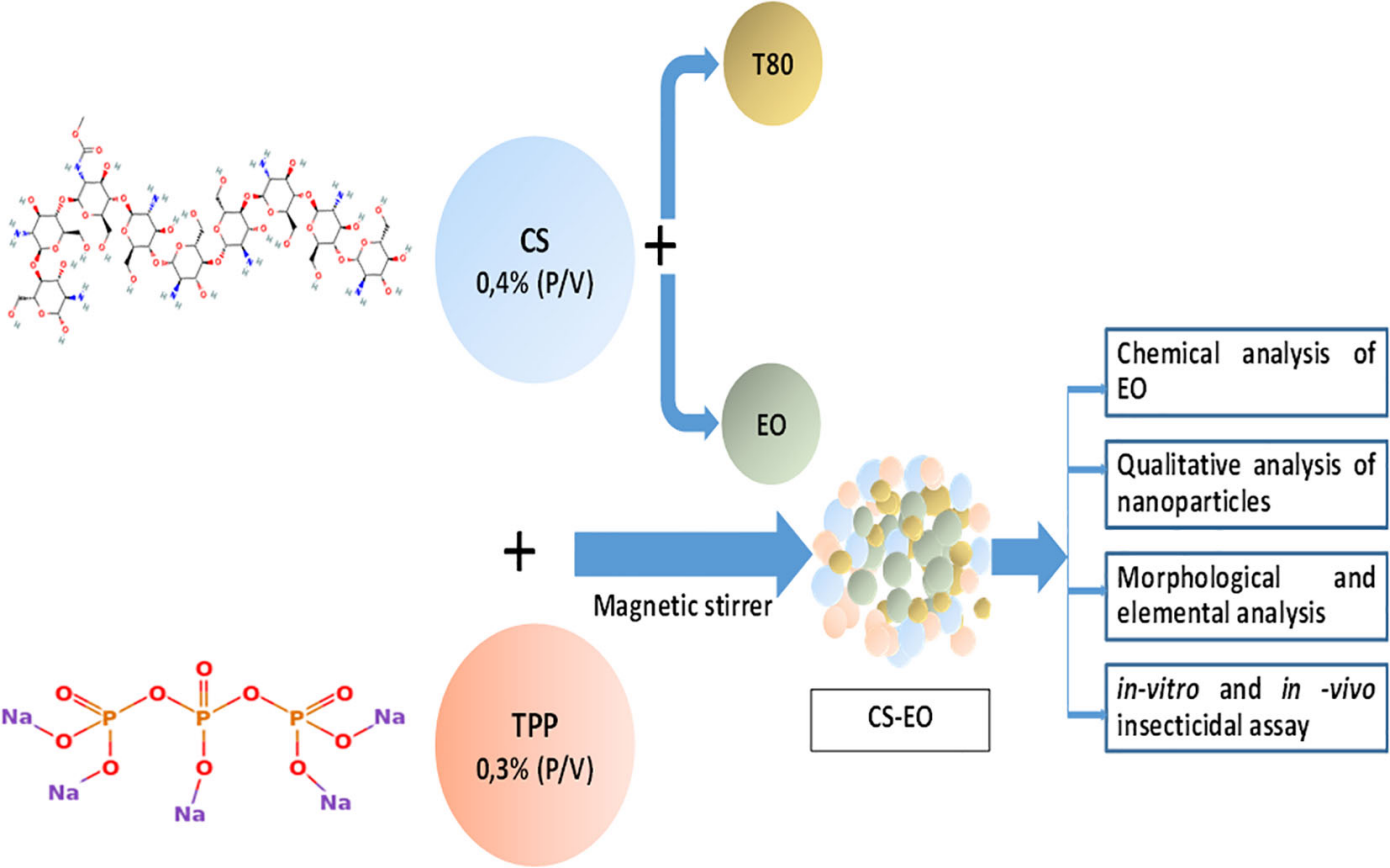
Schematic representation of the ionotropic gelation process used for chitosan nanoparticle synthesis encapsulating *H. italicum* essential oil. T80, Tween 80 emulsifier; CS, Chitosan; EO, Essential oil; TPP, sodium tripolyphosate, CS–EO, chitosan essential oil nanoparticle.

Sucrose (5% w/v) was added prior to lyophilization to protect nanoparticle integrity. Freeze-drying was performed using a Martin Christ Alpha 1-2 LD system (20Pa, −50°C, 24h) after cold centrifugation (20,000 rpm, 4°C, 30min) to remove unencapsulated oil. Control nanoparticles (without EO) were prepared identically (**Figure 1****).** All batches were stored in amber glass vials at 25°C with desiccant. The synthesis parameters included a CS/TPP mass ratio of 1.33:1, pH adjustment, and centrifugation force.

### 2.6 Quantitative analysis of nanoparticles

The nanoparticle production yield (Y %) was calculated as the ratio between the dry weight of freeze-dried nanoparticles and the total weight of raw materials initially used in the formulation, according to Equation 1:

(1) $$Y(\%)=\frac{W_{NP}}{W_{RM}}\times 100$$

Where *W_NP_* is the dry weight of freeze-dried nanoparticles (g) and *W_RM_* is the total weight of raw materials initially used (g).

This parameter reflects the conversion efficiency of the formulation process, indicating the proportion of starting substances successfully incorporated into the final nanoparticulate product.

Encapsulation performance was evaluated using UV–Vis spectroscopy at 275 nm. After centrifugation (20,000 × g, 30 min), the supernatant was analyzed to determine the concentration of non-encapsulated essential oil (EO). A calibration curve was established to quantify free EO, allowing indirect estimation of the encapsulated fraction by subtraction. Nanoparticles used for control (formulated without EO) were subjected to the same procedure to prevent spectral interference. Encapsulation efficiency (EE %) and payload loading (PL %) were calculated according to Equations 2, 3:

(2) $$EE(\%)=\;\frac{W_{EO,enc}}{W_{EO,tot}}\;\times 100$$

Where *W_EO_,enc* is the weight of encapsulated essential oil (g) and *W_EO_,tot* is the total weight of essential oil initially used (g).

(3) $$PL(\%)\;=\;\frac{w_{E0,enc}}{W_{NP}}\times 100$$

Where *W_EO_,enc* is the weight of encapsulated essential oil (g) and *W_NP_* is the dry weight of nanoparticles (g). All analyses were performed in triplicate to ensure data reliability.

The mean particle size and polydispersity index (PDI) of the nanoparticle formulations were determined using dynamic light scattering (DLS). The zeta potential, indicating surface charge, was measured by laser Doppler electrophoresis with a Beckman Coulter DelsaMax PRO analyzer. Prior to analysis, samples were diluted at a 1:10 volume ratio in ultrapure water and subjected to sonication (35 kHz, 5 min) to promote homogeneous dispersion. All measurements were carried out at 25.0 ± 0.1 °C and repeated three times to ensure reproducibility.

### 2.7 Structural characterization of nanoparticles

Structural characterization of the nanoparticles was conducted using Fourier-transform infrared (FTIR) spectroscopy (Agilent Cary 630) equipped with an attenuated total reflectance (ATR) module. Spectra were recorded in the range of 4000–400^-1^ cm with a spectral resolution of 4 cm. UV–visible spectroscopy (Agilent Cary series) was employed to verify compositional integrity, with absorbance measurements carried out in 2 mm quartz cuvettes. To ensure reproducibility, UV–Vis spectra were obtained under controlled humidity conditions, and batch-to-batch reproducibility was assessed by comparing absorbance values across replicates. All analyses were conducted at the Enzyme Catalysis Group laboratory, University of Southern Denmark.

The surface morphology and elemental composition of the nanoparticles were investigated using field emission scanning electron microscopy (FESEM, FEI Q250, Thermo Fisher Scientific), coupled with energy-dispersive X-ray spectroscopy (EDX). Samples were suspended in 0.1 M phosphate buffer (pH 7.4), deposited on aluminum stubs by drop-casting, and sputter-coated with gold (15 nm thickness) to enhance conductivity. SEM imaging was performed at accelerating voltages of 10, 15, and 20 kV to optimize image resolution while minimizing electron beam-induced degradation. Simultaneous elemental mapping by EDX provided complementary data regarding the composition of the nanoparticulate matrix.

### 2.8 Molecular docking studies

An *in silico* molecular docking approach was employed to predict the interaction between essential oil constituents and acetylcholinesterase (AChE). The three-dimensional structure of AChE from *Drosophila melanogaster* (PDB ID: 6XYU), complexed with the tacrine derivative 9-(3−iodobenzylamino)-1,2,3,4−tetrahydroacridine was retrieved from the Protein Data Bank. This structure, determined experimentally by X-ray diffraction at a resolution of 2.51Å, was preprocessed by removing crystallographic water molecules and native ligands. Hydrogen atoms were added, and partial charges were corrected to optimize molecular geometry. Ligand structures were obtained from the PubChem database and subjected to conformational optimization using Open Babel, before conversion to PDBQT format. Docking simulations were initially performed with the Molecular Operating Environment (MOE) software, followed by refinement using AutoGrid tools. The protein’s active site was identified, and a docking grid was constructed centered on this region. Resulting poses were visualized with Discovery Studio Visualizer (2021 edition), and interaction patterns were analyzed based on binding energy scores. This workflow adheres to established docking protocols widely applied in insect AChE studies (Arun et al., 2025). To further validate the reliability of the docking simulations, the root-mean-square deviation (RMSD) between predicted and reference ligand poses was calculated, confirming the consistency and accuracy of the binding predictions.

### 2.9 Insecticidal bioassays

#### 2.9.1 *In vitro* assay

Bioassays were conducted under controlled laboratory conditions (20–26°C; 60–70% relative humidity). Ten adult *A.* sp*iraecola* were gently transferred with a moistened brush onto citrus leaf discs placed on 1% nutrient agar in aerated Petri dishes to prevent desiccation. Essential oil (EO) solutions (10, 7, 4, and 1 µL/mL) were prepared in sterile distilled water containing 0.1% Tween 20 and applied uniformly with a mini-sprayer (0.25 mL per disc) (Cloyd et al., 2009). Mortality was recorded at 6, 24, 48, and 72 h post-treatment. Each condition was replicated four times, with controls including a positive control (W^+^, Acetamiprid 20%) and a negative control (W^-^, distilled water + Tween 20).

Nanoparticle bioassays (0.5, 0.25, and 0.125 mg/mL) were performed under the same conditions. Formulations were dispersed in distilled water, preheated to ensure homogeneity, and applied by direct contact to leaf discs. Each treatment was replicated three times. Aphids were considered dead when failing to respond to gentle probing. Acetamiprid 20% was used as a positive control, while unloaded chitosan nanoparticles (CS) served as the negative control.

#### 2.9.2 In semi-field assay

The second phase of the assay was performed *in vivo* under semi-field conditions using potted broad bean (*Vicia faba* L.) seedlings grown to the three-leaf stage. Leaves were artificially infested with 30 apterous adult *Aphis* sp*iraecola* per plant, transferred with a soft brush and allowed to settle for 24 h before treatment. *Helychrisum italicum* EOs were tested at four concentrations: LD_50_ from *in vitro* assays (≈8 µL/mL), half LD_50_ (≈4 µL/mL), double LD_50_ (≈17 µL/mL), and LD_90_ (≈16 µL/mL). Controls included a positive control (W^+^, Acetamiprid 20%) and a negative control (W^-^, distilled water + Tween 20). A total of 24 pots were arranged in a completely randomized block design, with 8 controls and 16 treated plants. Foliar spraying was applied uniformly, and plants were covered with fine mesh fabric to prevent aphid escape. Population assessments were conducted at 24, 48, and 72 h using a hand lens and fine needle. Each treatment was replicated four times under semi-controlled greenhouse conditions (20–26°C, 60–70% RH, 16:8 h photoperiod).

#### 2.9.3 Statistical analysis

Data were analyzed using R software (v4.2.1) (R Core Team, 2024). Normality was verified with the Shapiro–Wilk test and homogeneity of variances with Levene’s test. *In vitro* mortality data were corrected using Abbott’s formula (Abbott, 1925) to account for natural mortality in the negative control group, according to Equation 4:

(4) $$Corrected\;mortality\;(\%)=\frac{Observed\;mortality\;(\%)\;-\;Control\;mortality\;(\%)}{100-Control\;mortality\;(\%)}\times 100$$

Corrected data were analyzed by one-way ANOVA followed by Tukey’s HSD (α = 0.05). Probit regression was applied to estimate LC_50_ and LC_90_ values with 95% confidence intervals for *in vivo* assays, two-way ANOVA was performed when assumptions were met; otherwise, the non-parametric Kruskal–Wallis test was used. Dose–time–mortality relationships were assessed using generalized linear mixed models, with Bonferroni corrections applied. Residual diagnostics and effect size evaluation ensured robustness of statistical interpretation as represented in **Supplementary Figure 1**.

## 3 Results

### 3.1 Chemical composition of *H. italicum* essential oil

**Figure 2:**
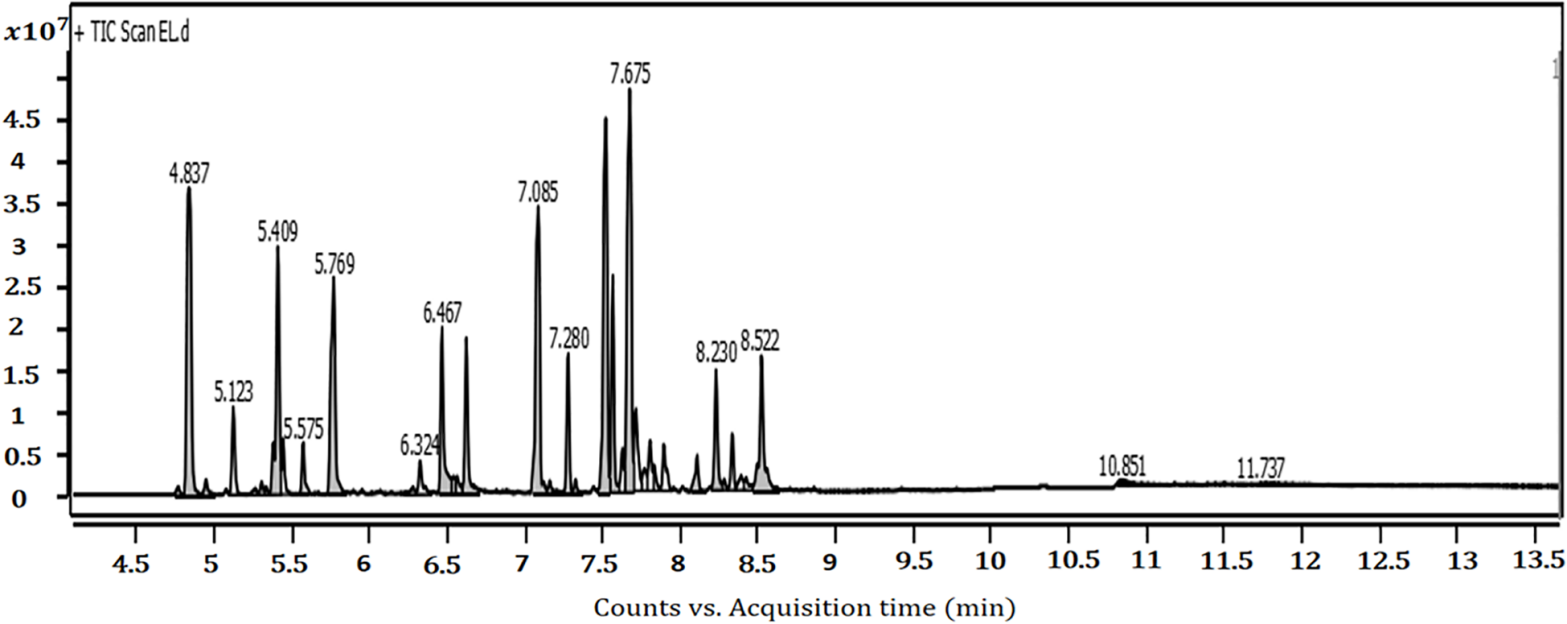
GC–MS chromatogram of *Helichrysum italicum* essential oil, showing the identified peaks corresponding to retention times (RT).

Gas chromatography–mass spectrometry (GC–MS) analysis identified 39 volatile compounds in the hydrodistilled essential oil (EO), representing 80.4% of the total composition (**Figure 2**, **Table 1**). The oil was characterized as a mixed chemotype rich in sesquiterpenes (27.39%) and oxygenated monoterpenes (25.18%). The dominant constituents were humulene (13.26%), caryophyllene (11.29%), and camphene (10.79%). Notable bioactive compounds included linalool (7.18%) and *cis*-geranyl acetate (9.16%).

**Table 1:** Chemical composition and class distribution of essential oil from aerial parts of *Helichrysum italicum* (North-central Algeria).

Compound	Area %	RT (min)	Molecular formula	Class	Class total %
Oxygenated Monoterpenes					25.177
Linalool	7.184	5.769	C10H18O	Monoterpene alcohol	
L-α-Terpineol	0.855	6.324	C10H18O	Monoterpene alcohol	
Cis-Géraniol	4.233	6.467	C10H18O	Monoterpene alcohol	
Benzoate de propyle	3.717	6.622	C10H12O2	Aromatic ester	
Acétate de cis-géranyl	9.158	7.085	C12H20O2	Monoterpene ester	
Sesquiterpenes					27.390
Copaene	2.844	7.280	C15H24	Sesquiterpene	
Humulene	13.255	7.675	C15H 24	Sesquiterpene	
Caryophyllene	11.291	7.520	C15H24	Sesquiterpene	
Monoterpenes					20.513
Camphène	10.786	4.837	C10H16	Monoterpene	
Sabinène	1.948	5.123	C10H16	Monoterpene	
D-Limonene	6.683	5.409	C10H16	Monoterpene	
γ-Terpinene	1.096	5.443	C10H16	Monoterpene	
Oxygenated Sesquiterpenes					7.323
Guaiol	2.828	8.230	C15H26O	Sesquiterpene alcohol	
Geranyl propionate	4.495	7.566	C13H22O2	Ester	
Total Identified	80.403				

RT, Retention time (mn).

This profile from the Algerian Blidean Atlas region showed distinct differences from Mediterranean and Central European chemotypes, particularly in its high camphene and humulene content.

### 3.2 Synthesis and physicochemical characterization of nanoformulations

Ionotropic gelation produced stable chitosan nanoparticles with high efficiency (**Table 2**). EO-loaded nanoparticles (CS-HI) exhibited a significantly higher yield (Y = 94.0%, *p* < 0.05) compared to blank CS nanoparticles (Y = 86.9%), Encapsulation efficiency (EE) reached 97.9 ± 1.2%, with payload loading (PL) of 20.9 ± 0.8%. Triplicate measurements confirmed these values. Centrifugation speed was critical: below 20,000 × g particles remained suspended, whereas speeds above 25,000 × g caused pellet compaction. Overall, yields (Y) exceeded 85%, demonstrating excellent retention of polymer and bioactive components as shown in **Supplementary Table 1**.

**Table 2:** Physicochemical properties of chitosan nanoparticles: Comparative analysis of *H. italicum* essential oil-loaded (CS–HI) and unloaded (CS) formulations.

NP	Size (nm) Mean	PDI	%Y	%PL	EE%
CS-HI	173.38	0.27	94.00	20.97%	97.85%
CS	90.20	0.55	86.95	–	–

NP, Nanoparticles; PDI: polydispersity index; Y, Yield; PL, payload loading; EE, encapsulation efficiency.

**Figure 3:**
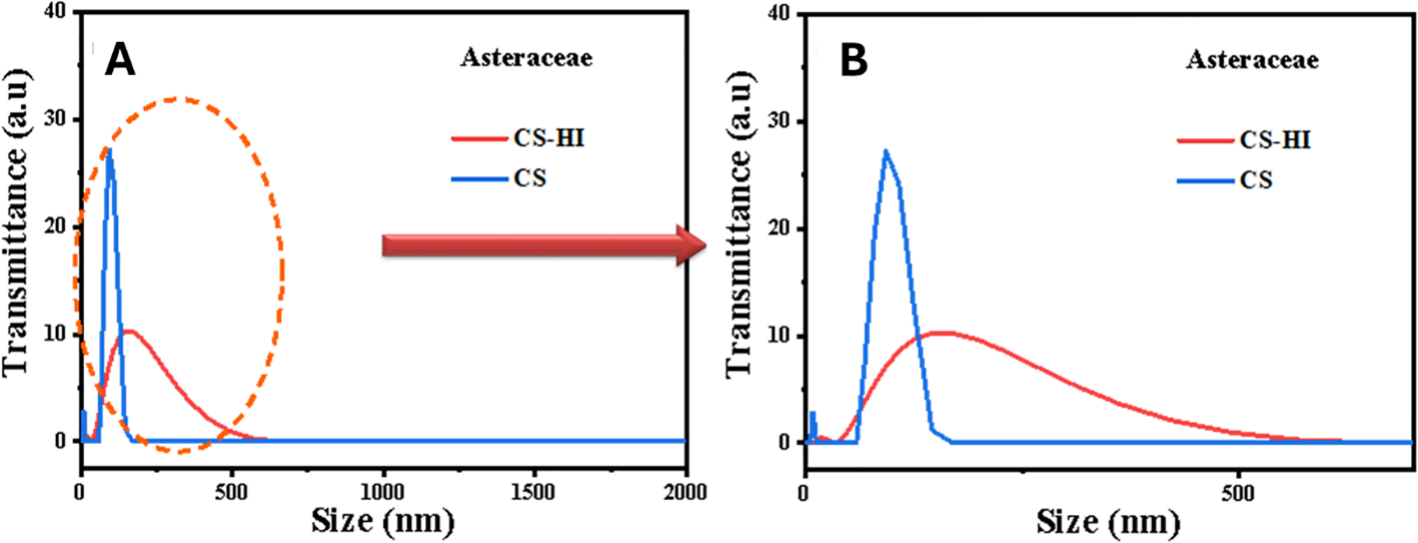
Size distribution profiles of *Helichrysum italicum* essential oil–loaded chitosan nanoparticles (CS–HI) compared to unloaded control (CS). UV-Vis absorbance spectra of chitosan nanoparticles (CS) and Helichrysum italicum essential oil-loaded chitosan nanoparticles (CS–HI): **(A)** full wavelength range (200-800 nm) and **(B)** detailed UV region (200‒300 nm) highlighting essential oil absorption characteristics.

Dynamic light scattering (DLS) revealed that EO-loaded CS–HI nanoparticles exhibited a mean hydrodynamic diameter of 173.4 ± 2.1 nm with low polydispersity (PDI = 0.27 ± 0.02), indicating a monodisperse population (**Figure 3**). In contrast, blank CS nanoparticles were smaller (90.2 nm) but more polydisperse (PDI = 0.55). Zeta potential values were highly positive for both formulations (+45.5 ± 1.3 mV for CS–HI and +46.1 ± 1.5 mV for CS), confirming excellent colloidal stability (**Table 3**).

**Table 3:** Surface charge characteristics and stability assessment of chitosan nanoparticles: comparison between *H. italicum*–loaded (CS–HI) and unloaded (CS) formulations.

Features	45.49 mV (CS-HI)	46.12 mV (CS)
Nature	Positive	Positive
Stability	Very high	Very high
Interaction	Attraction Charges (+)	Neutral interaction
Application	Favorable	Good stability

These findings highlight the robustness of the ionotropic gelation process and the suitability of CS–HI nanoparticles for efficient EO delivery.

### 3.3 Spectroscopic, morphological, and elemental analysis

**Figure 4:**
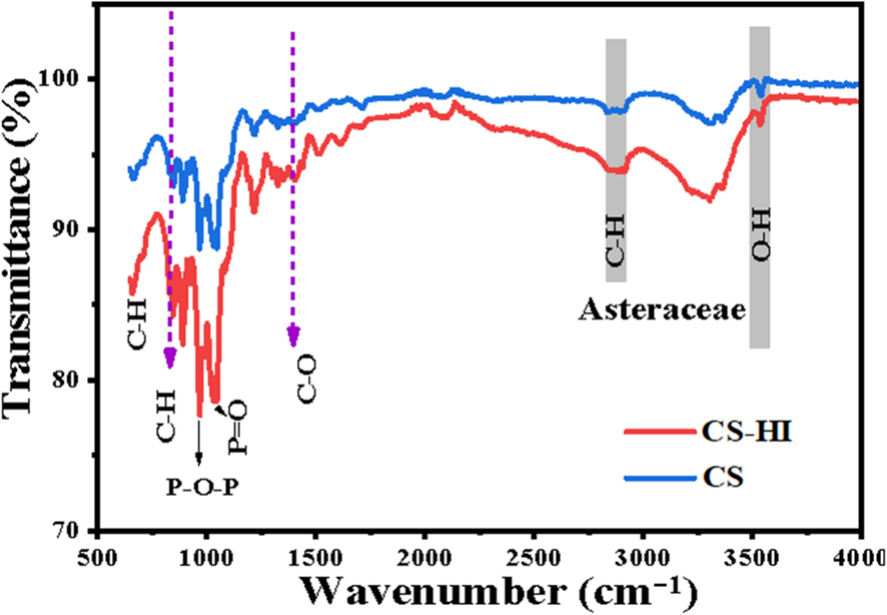
FTIR spectral comparison of *Helichrysum italicum* essential oil–loaded chitosan nanoparticles (CS–HI) versus unloaded control (CS).

FTIR spectroscopy (400–4000^-^¹ cm, 4 cm resolution) confirmed successful encapsulation, showing a 15 cm red shift in the carbonyl stretch (to 1740 cm) and additional bands consistent with chitosan–TPP crosslinking (**Figure 4**, **Table 4**).

**Table 4:** FTIR vibrational assignments and functional group analysis of *H. italicum*–loaded chitosan nanoparticles (CS–HI) versus unloaded control (CS).

Absorption peak (cm^-^¹)	Transmittance (%)	Bond/Vibration	Functional group	Sample presence
650−675	70−90	C-H stretch	Aromatic	CS-HI > CS
810−830	60−85	C-H bend	Aromatic	CS-HI only
930−950	50−73	P-O/C-O-C	TPP crosslink	Both
970−980	–	P-O-P	TPP	Both
1020	–	P=O/C-O-C	Phosphate/Polysaccharide	Both
1250	–	C-O/P-O	Ester/Phosphate	CS-HI > CS
1375	–	C-O	Carboxylate	CS-HI only
1460	–	C-H bend	Methyl	CS-HI only
1740	–	C=O stretch	Ester	CS-HI only
2850−2875	–	C-H stretch	Aliphatic (-CH_2_, -CH_3_)	CS-HI > CS
2920	–	C-H stretch	Aliphatic chains	CS-HI > CS
3000−3400	–	O-H stretch	Hydroxyl/Carboxylic acid	Both
3275−3525	–	O-H stretch	Free hydroxyl	Both

TPP, tripolyphosphate (crosslinking agent).

**Figure 5:**
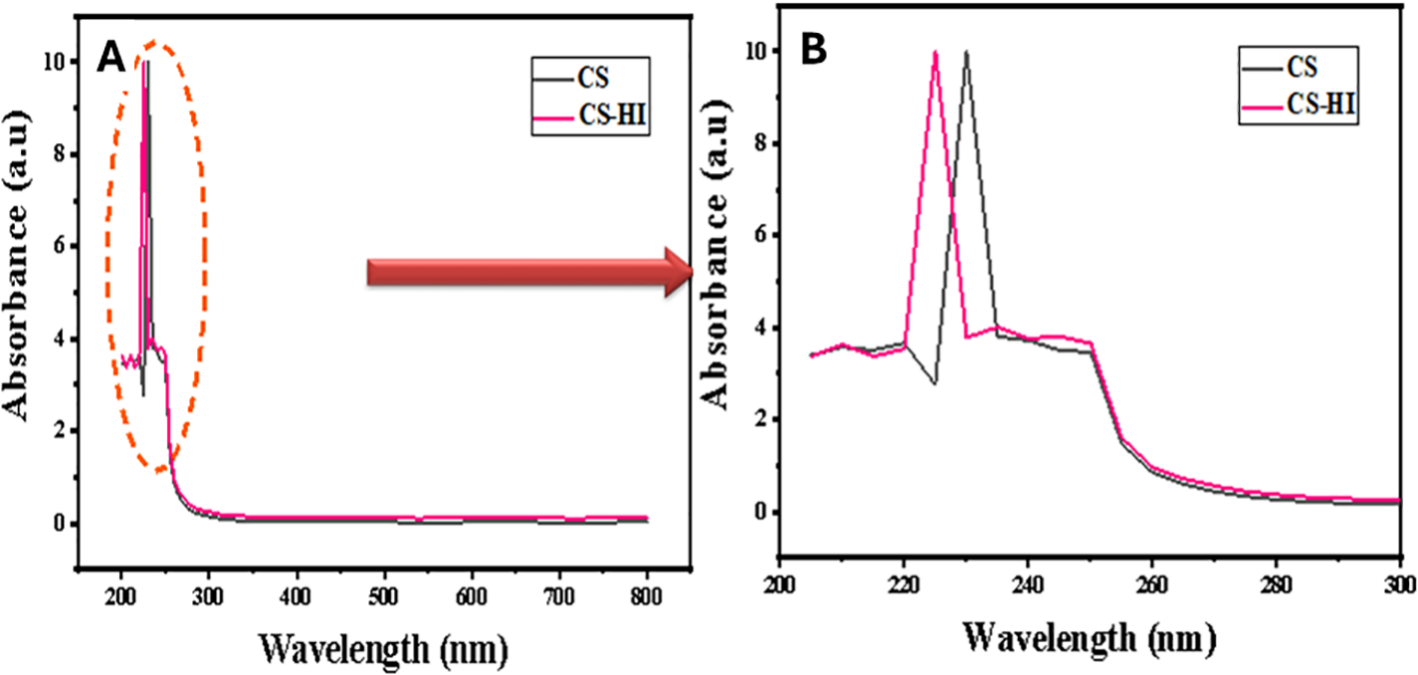
UV-Vis absorbance spectra of chitosan nanoparticles (CS) and *Helichrysum italicum* essential oil-loaded chitosan nanoparticles (CS–HI): **(A)** full wavelength range (200–800 nm) and **(B)** detailed UV region (200–300 nm) highlighting essential oil absorption characteristics.

UV–Vis analysis revealed minimal batch-to-batch variation (<5% absorbance difference), underscoring the importance of humidity control for reproducible amide band detection. UV–Vis spectroscopy showed a characteristic absorption peak was observed at 220–230 nm for CS–HI, absent in the control, indicating the presence of phenolic compounds from the essential oil (**Figure 5**).

### 3.4 Morphological analysis by FESEM

**Figure 6:**
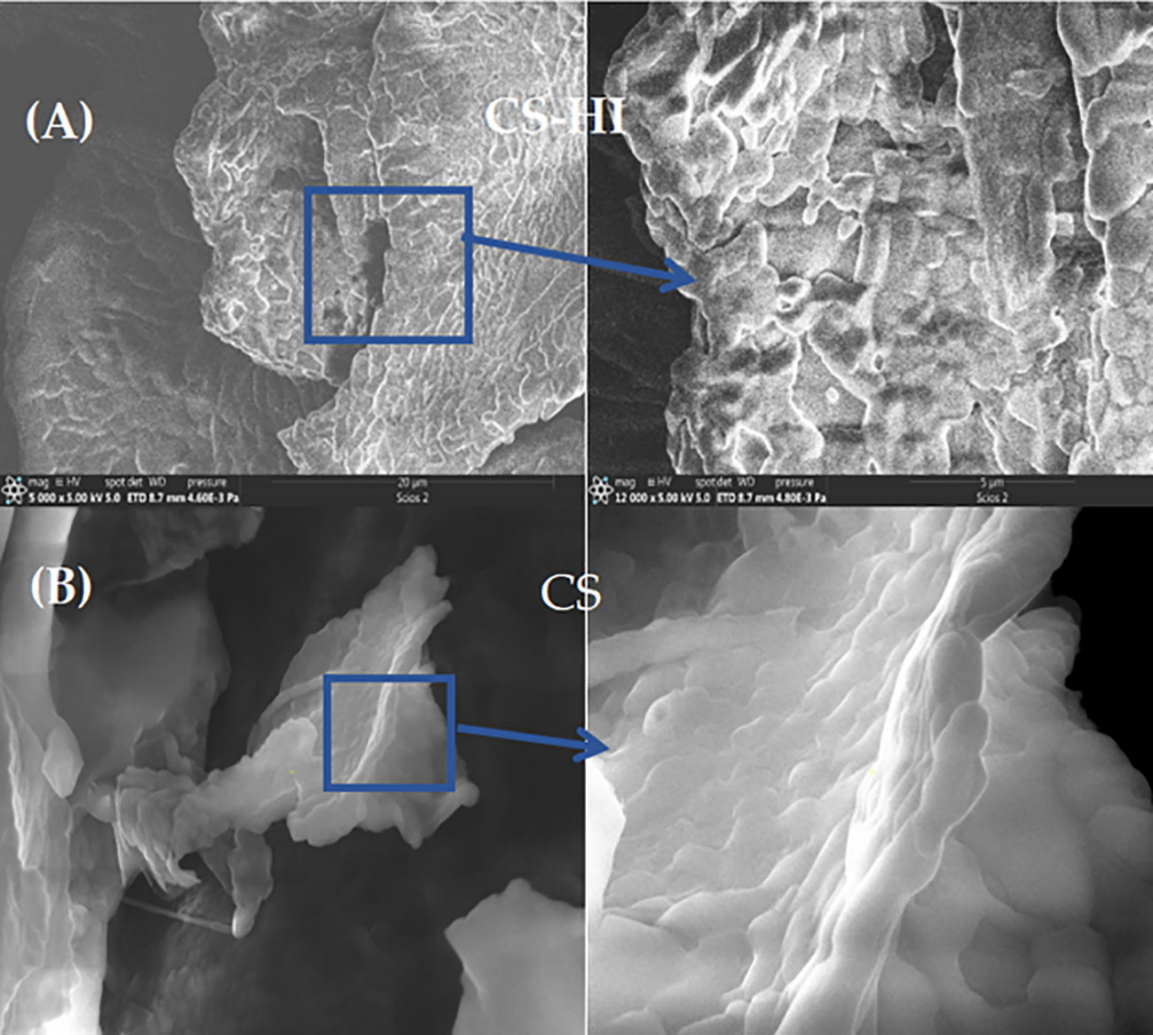
**(A)** Morphological comparison of *Helichrysum italicum* essential oil–loaded chitosan nanoparticles (CS–HI) and **(B)** unloaded control (CS) via FESEM. The blue square shows the magnified area.

Field emission scanning electron microscopy (FESEM) revealed spherical morphology with smooth surfaces for CS–HI nanoparticles (**Figure 6**), whereas blank CS nanoparticles displayed irregular shapes. FESEM imaging performed at 15 kV on gold-sputtered samples confirmed the presence of spherical nanoparticles with an average diameter of ~170 nm and smooth surfaces.

### 3.5 Elemental analysis by EDX

**Figure 7:**
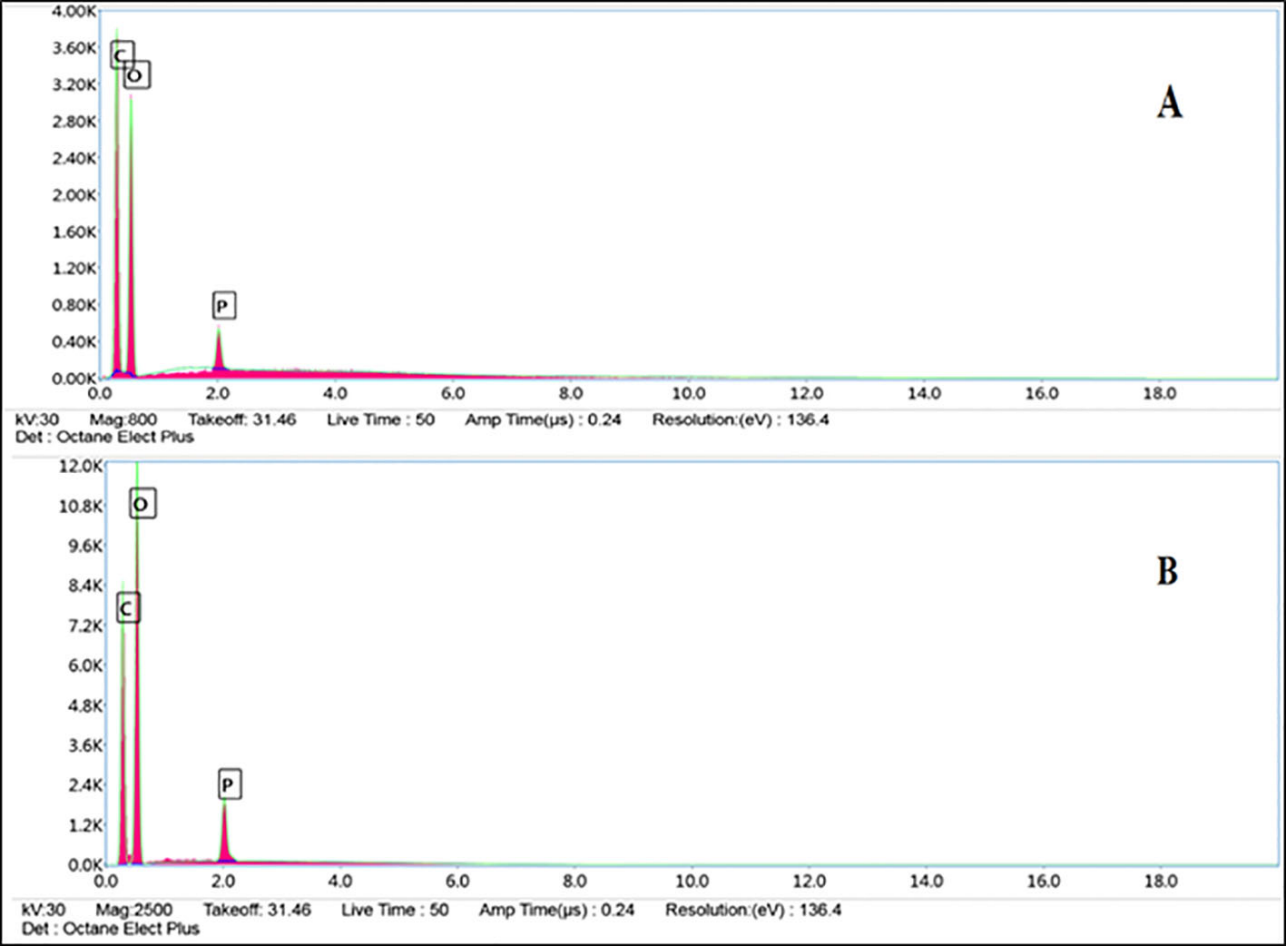
EDX spectral comparison of *Helichrysum italicum* essential oil–loaded chitosan nanoparticles (CS–HI) **(A)** and unloaded control (CS) **(B)**.

Energy-dispersive X-ray (EDX) analysis confirmed TPP crosslinking through phosphorus detection (0.48 wt %) and provided direct evidence of EO encapsulation. CS–HI nanoparticles exhibited markedly higher carbon content (58.95 wt %) compared to blank CS nanoparticles (41.53 wt %), consistent with the incorporation of essential oil components (**Figure 7**; **Table 5**).

**Table 5:** Comparative elemental composition (wt %) of *H. italicum* essential oil–loaded chitosan nanoparticles (CS–HI) and unloaded control (CS) from EDX analysis.

Element	CS-HI (wt%)	CS (wt%)	Significance
C	58.95	41.53	↑57% in CS-HI (essential oil hydrocarbons)
O	40.57	57.05	↓29% in CS-HI (dilution effect)
P	0.48	1.41	Consistent crosslinking (TPP residues)
C/O ratio	1.45	0.73	Confirms organic-polysaccharide hybrid matrix

### 3.6 Molecular docking analysis

**Figure 8:**
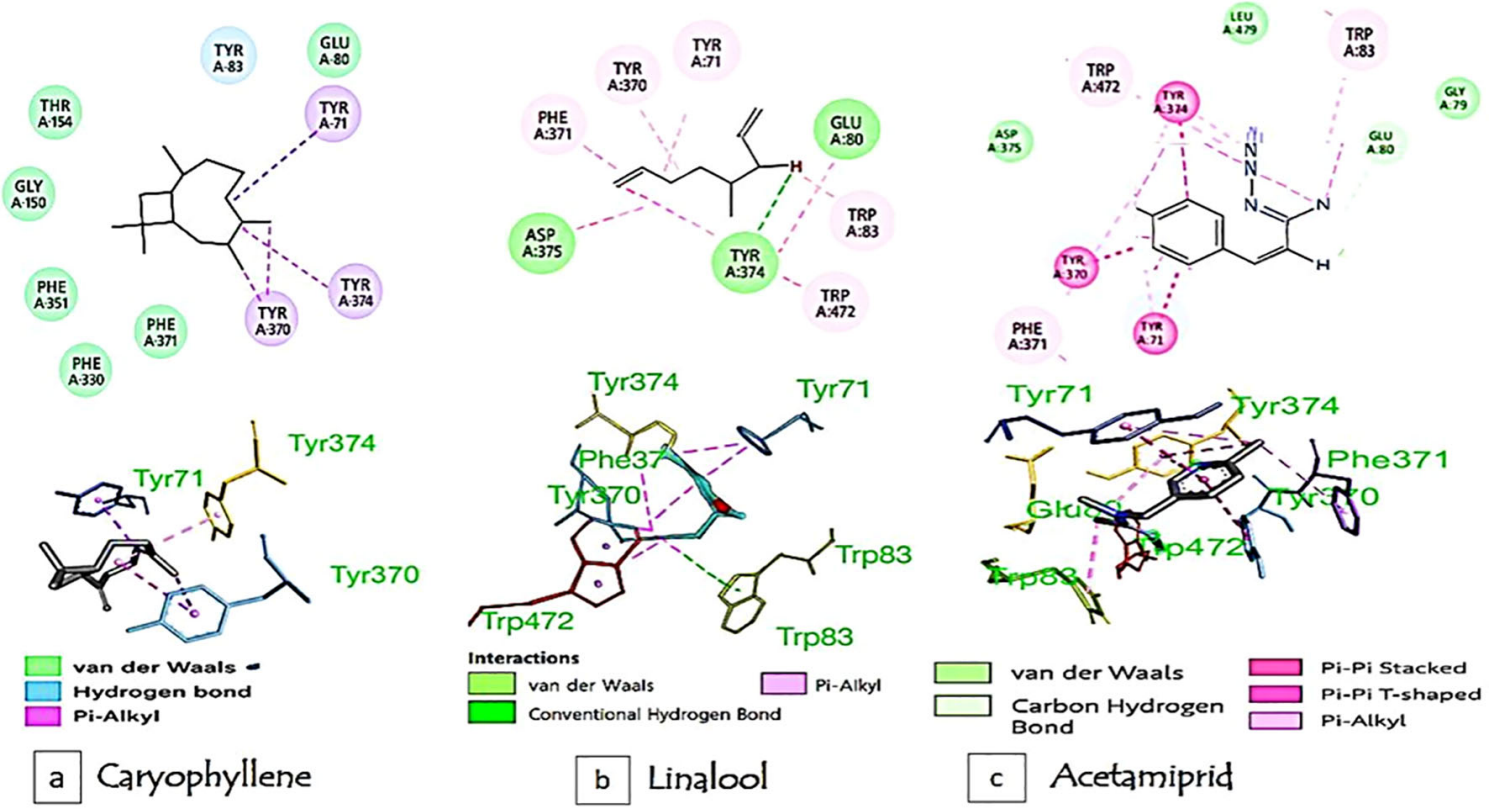
Comparative molecular docking analysis of acetamiprid, linalool, and caryophyllene binding to aphid acetylcholinesterase (AChE). Comparative molecular docking analysis of **(a)** caryophyllene, **(b)** linalool, and **(c)** acetamiprid binding to aphid acetylcholinesterase (AChE).

Molecular docking simulations (PDB ID: 6XYU) confirmed strong binding of major EO constituents to aphid acetylcholinesterase (AChE). Caryophyllene exhibited the highest affinity (–7.10 Kcal/mol) with stable conformations, forming hydrophobic interactions with Trp83, Phe371, and Tyr residues in the active site. Linalool showed moderate binding (–5.64 Kcal/mol), primarily through hydrogen bonding with Tyr370. These interactions align with *in vitro* AChE inhibition results and suggest a competitive inhibition mechanism. As a positive control, acetamiprid displayed the strongest binding (–7.74 Kcal/mol) but with high conformational flexibility (**Figure 8**; **Table 6**).

**Table 6:** Thermodynamic and structural parameters from molecular docking analysis of acetamiprid, caryophyllene, and linalool binding to aphid acetylcholinesterase (AChE).

Compound	Binding energy (kcalmol^-^¹)	Inhibition constant (µM)	Internal energy (kcalmol^-^¹)	Entropy (kcalmol^-^¹K^-^¹)	Characteristics
Linalool	-5.64	73.41	-4.97	4.58	High conformational flexibility, suitable as synergist. ^47,48^
Caryophyllene	-7.10	6.27	0.00	0.00	Optimal stability/specificity balance; no entropic cost. ^49,50^
Acetamiprid	-7.74	2.11	-7.11	4.58	Strongest inhibition but high conformational instability.^51^

**Figure 9:**
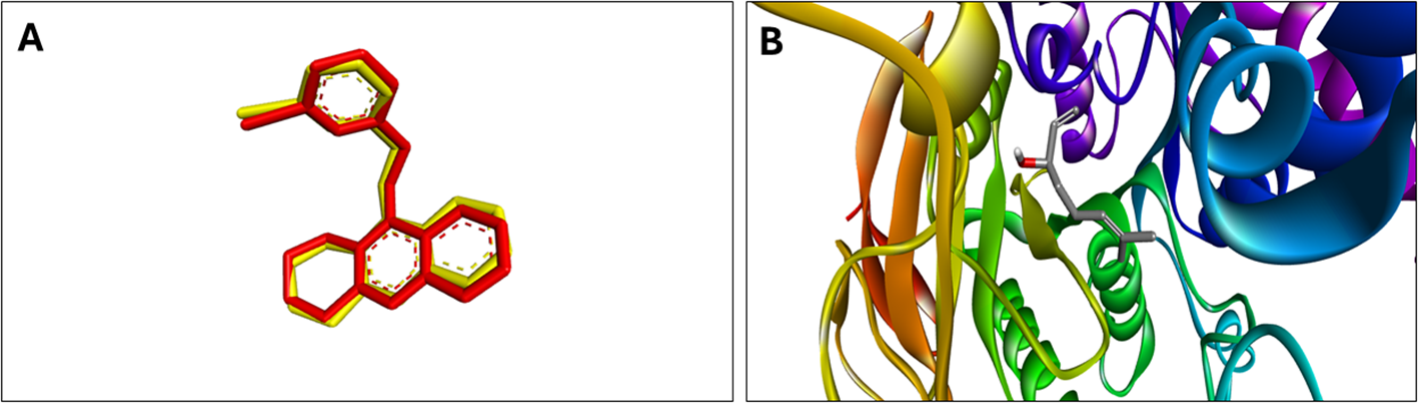
Structural and dynamic validation of the ligand–AChE complex. **(A)** Root-mean-square deviation (RMSD) analysis confirming docking accuracy and stability of the ligand–AChE complex. **(B)** Structural visualization of the ligand bound within the active site of AChE, highlighting key binding interactions.

An RMSD value of 0.36Å was obtained for the ligand–AChE complex, demonstrating excellent agreement with the reference pose and thereby validating the docking protocol (**Figure 9**).

### 3.7 Insecticidal efficacy of essential oil and nanoformulations

#### 3.7.1 *In vitro* bioassay of essential oil

Under these experimental conditions, the efficacy of different doses of *H. italicum* essential oil (1, 4, 7, and 10 µL/mL) was compared with the negative control (distilled water + Tween 80) and the positive control (Acetamiprid 20). Mortality and behavioral symptoms were assessed at 6, 12, 24, and 48 h, revealing significant differences among treatments.

**Figure 10:**
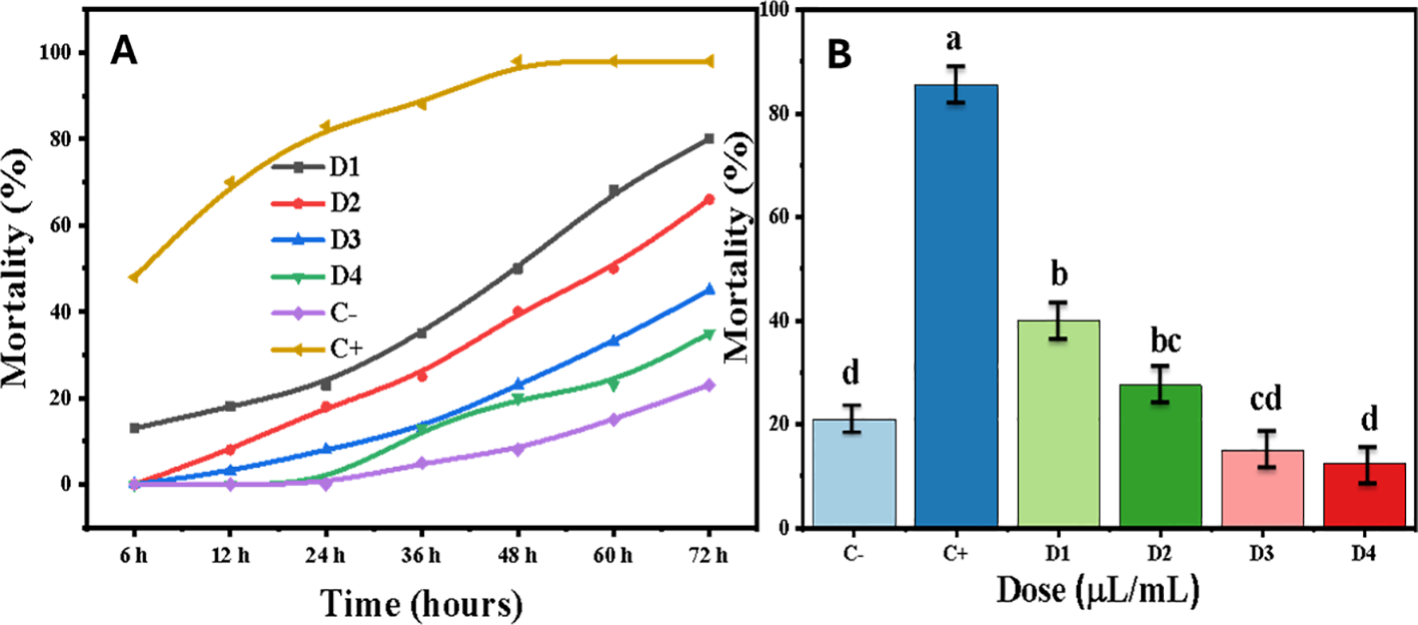
**(A)** Temporal mortality patterns of *Aphis* sp*iraecola* under graded *Helichrysum italicum* essential oil exposure and **(B)** dose–response mortality showing concentration- and time-dependent effects (*in vitro* bioassay; Tukey’s HSD). Treatments sharing the same lower case letter (a, b, c, d) are not significantly different from each other, while treatments with different letters differ significantly at *p* < 0.05.

The essential oil of *H. italicum* exhibited dose- and time-dependent toxicity against *A.* sp*iraecola* (**Figure 10**). At 72 hours after treatment, the highest concentration (10 µL/mL) caused 80% mortality.

Lethal dose calculations indicated a reduction in LC_50_ values from 8.26 µL/mL at 60 h post-treatment (HPT) to 6.35 µL/mL at 72 HPT (**Table 7**). A biphasic response was evident, characterized by a latency phase between 12–24 h, followed by a sharp increase in mortality. As shown in **Supplementary Figure 2**.

**Table 7:** Statistical analysis of *H. italicum* essential oil toxicity against *A. spiraecola*: ANOVA results and lethal dose calculations.

Analysis type	Time (h)	LD_50_ (µL mL^-1^)	LD_90_ (µL mL^-1^)	Regression	R²	F-value	*p*-value
One-way ANOVA	–	–	–	–	–	95.36	2.62×10^-^¹²
Lethal dose	60	8.26	14.97	Y =0.75 + 5.96x	0.985	–	–
Lethal dose	72	6.35	12.20	Y=6.53 + 6.84x	0.977	–	–

LD_50_, median lethal dose; LD_90_, lethal dose for 90% mortality; R², coefficient of determination; F-value.

Fisher’s statistic; *p*-value, probability value.

#### 3.7.2 Semi-field (*in vivo*) bioassay of essential oil

**Figure 11:**
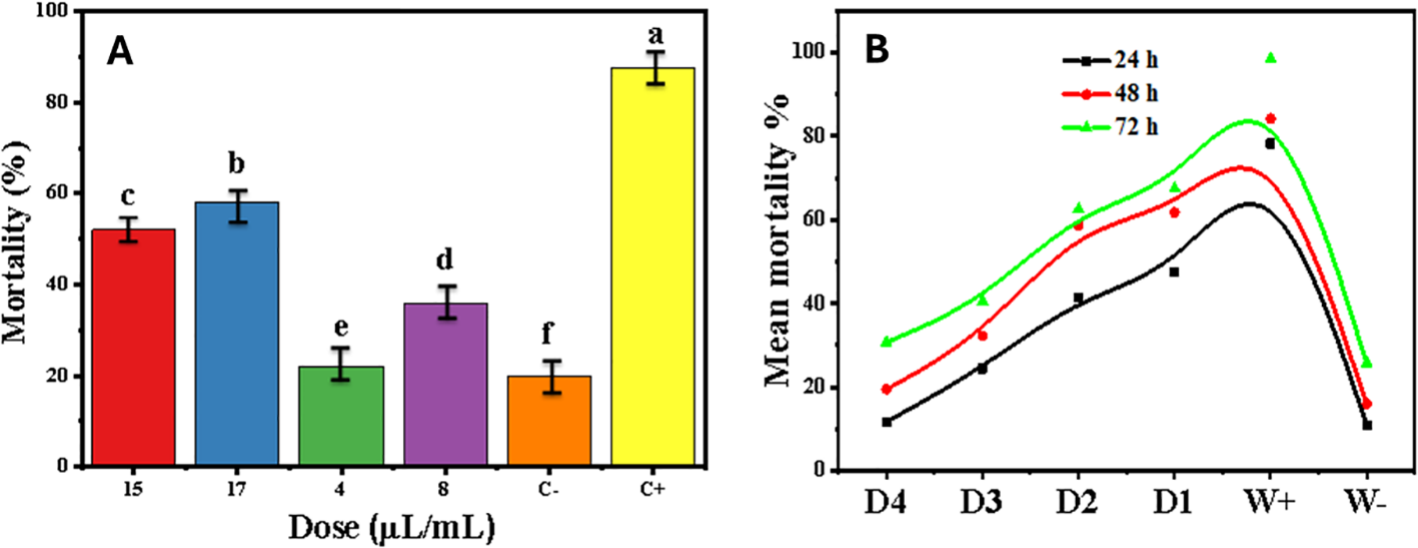
Dose-dependent efficacy and temporal mortality kinetics of *H. italicum* essential oil against *A.* sp*iraecola* under semi-field conditions. **(A)** Dose–response mortality at 72 h post-treatment and **(B)** time-course mortality at different concentrations of *H. italicum* essential oil. Treatments sharing the same lower case letter (a, b, c, d, f) are not significantly different from each other, while treatments with different letters differ significantly at *p* < 0.05.

Under semi-field conditions, the efficacy of *H. italicum* essential oil was reduced compared to laboratory assays (**Figure 11**). At 72 h post-treatment (HPT), the highest concentration (17 µL/mL) resulted in 67% mortality. The LD_50_ increased to 10.91 µL/mL, while the LD_90_ reached 28.00 µL/mL, reflecting a marked reduction in potency attributable to environmental factors (**Table 8**). Additional characterization data are provided in **Supplementary Figure 2**.

**Table 8:** Statistical parameters and lethal dose (LD) progression of *H. italicum* essential oil against *A. spiraecola* under semi-field conditions.

Analysis type	Time (h)	LD_50_ (µLmL^-^¹)	LD_90_ (µLmL^-^¹)	Regression equation	R²	F-value	*p*-value	Significance
Two-way ANOVA	**-**	**-**	**-**	**-**	**-**	1221.978*	<2×10^-^¹^6^	(*p*< 0.001)***
(Dose effect)	**-**	**-**	**-**	**-**	**-**	295.117*	<2×10^-^¹^6^	***
(Time effect)						4.307‡	0.000195	***
Lethal dose	48	13.18	27.20	Y=0.0791x-1.204x	0.421	**-**	**-**	**-**
Lethal dose	72	10.91	28.00	Y=0.091x-1.077x	0.427	**-**	**-**	**-**

Dose (df = 5); Time (df = 2); Dose × Time (df = 10); Residuals (df = 54).LD_50_ corresponds to the dose that reduce the population 50%.LD_90_ corresponds to the dose that reduce the population 90%.

A two-way ANOVA revealed highly significant effects of both dose and exposure time (p < 2 × 10^-^¹^6^). Semi-field trials conducted on *V. faba* demonstrated 20–30% lower efficacy than *in vitro* tests, likely due to environmental degradation.

#### 3.7.3 *In vitro* bioassay of nanoencapsulated formulation (CS–HI)

**Figure 12:**
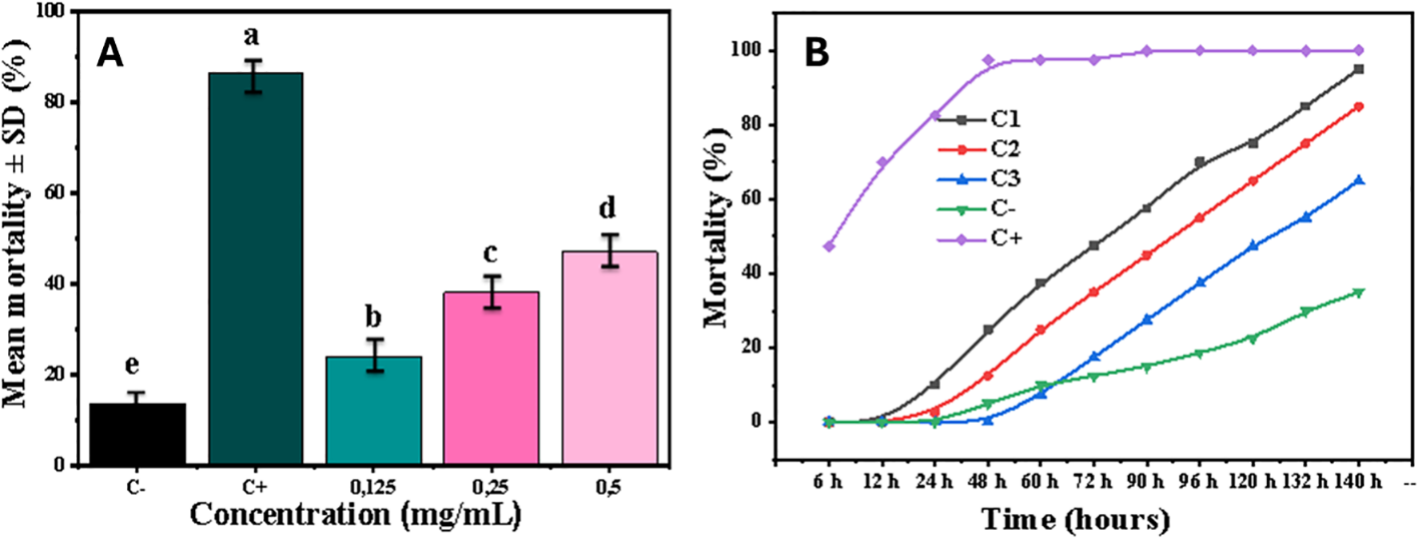
*In vitro* bioactivity of chitosan-encapsulated *Helichrysum italicum* essential oil (CS–HI) against *Aphis* sp*iraecola*. **(A)** Dose–response efficacy of CS–HI against aphids and **(B)** time–mortality kinetics of CS–HI over 140 h post-treatment. Treatments sharing the same lower case letter (a, b, c, d) are not significantly different from each other, while treatments with different letters differ significantly at *p* < 0.05.

Nanoencapsulation markedly enhanced and prolonged insecticidal activity (**Figure 12**). The CS–HI formulation at 0.5 mg/mL achieved 100% mortality by 140 h post-treatment (HPT). The LD_50_ decreased from 0.48 mg/mL at 90 HPT to 0.35 mg/mL at 96 HPT. Additional characterization data are provided in the Supporting Information (**Supplementary Figures 3**, **4**), demonstrating time-dependent cumulative toxicity (**Table 9**). The formulation exhibited a controlled-release profile, with a 24–48 h latency period followed by rapid escalation in efficacy. A one-way ANOVA confirmed a highly significant dose effect (F (4.15) = 259.2, *p* = 1.2 ×10^-^¹³).

**Table 9:** Combined statistical analysis of CS-HI nanoparticle efficacy against *A. spiraecola*.

Analysis type	Time (h)	LD_50_ (mg mL^-^¹)	LD_90_ (mg mL^-^¹)	Regression equation	R²	F-value	*p*-value	Significance
One-way ANOVA	–	–	–	–	–	259.2 (4.15)	1.2×10^-^¹³	***
Lethal concentration	90	0.48	2.31	Y=7.35 + 89.09x	0.92	–	–	–
Lethal concentration	96	0.35	0.74	Y=13.87 + 102.39x	0.99	–	–	–

ANOVA parameters: df = 4 (concentration); df = 15 (residuals); ***p < 0.001.LD_50_ corresponds to the dose that reduce the population 50 %LD_90_ corresponds to the dose that reduce the population 90%.

## 4 Discussion

### 4.1 Chemical profile and ecological relevance

The essential oil (EO) of *Helichrysum italicum* obtained under Mediterranean microclimates displayed a distinctive composition, marked by unusually high camphene (10.79%) and the presence of propyl benzoate (3.72%). Such compounds, rarely reported in European populations (Mollova et al., 2020; Węglarz et al., 2022), highlight the metabolic plasticity of the species and may reflect ecophysiological adaptations to thermal or water stress. The co-occurrence of camphene, caryophyllene, and terpineol suggests synergistic effects, reinforcing insecticidal activity at low concentrations, as observed in other multi-component essential oils (Pavela and Benelli, 2016). In contrast to the neryl acetate–dominated profiles reported in Central Europe, our oil exhibits a stable sesquiterpene signature (Mollova et al., 2020; Ninčević et al., 2019; Węglarz et al., 2022), advantageous for formulation and persistence under semi-field conditions. This chemical diversity, by mitigating the risk of resistance, underscores the relevance of *H. italicum* EO in integrated pest management strategies (Aioub et al., 2024; Ayllón-Gutiérrez et al., 2024; Bourenane et al., 2025).

### 4.2 Nanoencapsulation performance and validation

Ionotropic gelation proved highly effective for EO nanoencapsulation, achieving exceptional encapsulation efficiency (97.85%) and consistently high production yields (>85%). EO-loaded CS–HI nanoparticles reached a 94% yield, markedly higher than unloaded CS controls (86.95%), suggesting that EO components actively stabilize the chitosan matrix during formation. Encapsulation improved particle uniformity (PDI 0.27 vs. 0.55 for CS alone) while maintaining optimal nanoparticle size (~173 nm), thereby balancing colloidal stability with biointeraction potential. The strong positive zeta potential (>+45 mV) further supports agricultural applicability by promoting adhesion to negatively charged insect cuticles and plant surfaces (Abd El-Monaem et al., 2022; Benamer Oudih et al., 2023; Hoang et al., 2022) . Each gram of nanoparticles delivered nearly 210 mg of active compounds (PL 20.9%), reducing carrier requirements and formulation costs in field applications. Such high encapsulation efficiencies are seldom reported for essential oils (Yammine et al., 2024), underscoring the technical advance represented by our approach. These findings are consistent with analogous reports on lipid–polymer hybrid nanoparticles (Hassan et al., 2025) and reinforce the importance of size and charge in governing nanoparticle interactions (Porfiryeva et al., 2025). Finally, given the limited research on ionic gelation for pest and pathogen control, our results highlight both the novelty and the practical relevance of this strategy in agricultural nanotechnology (Ghorai et al., 2025).

Spectroscopic and elemental analyses (FTIR, UV–Vis, EDX) provided convergent evidence of successful EO incorporation and stable chitosan–TPP crosslinking in line with previous reports on chitosan nanoparticles produced by ionic gelation (Alehosseini et al., 2022). The red shift of the carbonyl stretch (1755 → 1740 cm) observed in FTIR spectra indicates hydrogen bonding between EO ester groups and chitosan hydroxyl moieties, a phenomenon rarely reported in essential oil encapsulation systems and consistent with observations on cross-linked chitosan films (Lewandowska, 2026). UV–Vis spectra revealed a distinct absorption peak at 220–230 nm, attributed to π→π transitions of phenolic and flavonoid constituents, thereby establishing a molecular fingerprint for EO presence. Similar absorption features have been reported in films enriched with polyphenols (Qamar et al., 2026). FESEM confirmed spherical morphology with smooth surfaces for CS–HI nanoparticles, a feature closely associated with improved bioavailability and controlled release of active compounds. Comparable observations have been reported for green-synthesized nanoparticles, where morphology plays a critical role in functional performance (Kazemi et al., 2023). EDX analysis further validated EO incorporation through increased carbon content (58.95% vs. 41.53% in CS) and a higher C/O ratio (1.45 vs. 0.73), consistent with the hydrocarbon-rich terpenoid profile identified by GC–MS and in agreement with reports on terpenoid-rich extracts obtained by supercritical CO_2_ extraction (Chen et al., 2024). Collectively, these complementary findings not only validate the structural integrity of CS–HI nanoparticles but also establish reliable analytical markers for quality control and scalability in agricultural applications.

### 4.3 Mechanistic insights from molecular docking

Molecular docking provided mechanistic support for the observed bioactivity of *H. italicum* EO constituents against aphid acetylcholinesterase (AChE). Caryophyllene exhibited strong binding affinity (−7.10 kcal/mol) and stable interactions with conserved residues in the active site, consistent with a competitive inhibition mechanism. Its bicyclic structure enabled more optimal binding pocket occupancy than linalool’s linear chain, rationalizing its superior efficacy in bioassays. Entropy values further revealed caryophyllene’s thermodynamic advantage over both acetamiprid and linalool, supporting its potential as a stable botanical alternative (Francomano et al., 2019; Gyrdymova and Rubtsova, 2022; Lisnasari et al., 2022; Singh and Mishra, 2024). These findings are in line with *in silico* studies highlighting the structural rigidity of β-caryophyllene oxide, which promotes stable interactions and reduces entropic penalties compared to more flexible ligands (Kamal, 2022). Although acetamiprid displayed slightly higher binding affinity (−7.74 kcal/mol), its conformational flexibility may compromise stability, making natural ligands such as caryophyllene competitive. This interpretation is consistent with reports of molecular disruption and selective toxicity induced by acetamiprid in beneficial insects (Ansari et al., 2026). Importantly, the EO does not act through a single compound but through a synergistic combination of caryophyllene, linalool, and other terpenoids. This multi-component mechanism enhances robustness and reduces the risk of resistance, consistent with reports on essential oils and microencapsulation strategies against biofilms and pathogens (Bustillos et al., 2025). Collectively, these quantitative differences in molecular interaction profiles explain the observed bioactivity patterns and highlight the multi-target synergistic potential of *H. italicum* EO as a sustainable botanical alternative to synthetic insecticides.

### 4.4 Bioassays and release kinetics

Insecticidal bioassays demonstrated the superior and prolonged toxicity of CS–HI nanoparticles compared to free EO, which suffered a 28% reduction in semi-field efficacy. The 2.2-fold lower LC_50_ of CS–HI and its sustained release profile leading to 100% mortality within 140 h highlight the reservoir function of chitosan in protecting volatile constituents. All EO treatments exhibited a latency phase (~12 h) before mortality sharply increased, consistent with sesquiterpene penetration dynamics (Gyrdymova and Rubtsova, 2022). This biphasic pattern reflects a multi-target mechanism (EFSA et al., 2024; Hernandez-Jerez et al., 2024) involving both acetylcholinesterase inhibition and octopamine receptor modulation, while the escalation period (12–62 h) required for maximal efficacy aligns with progressive bioaccumulation in terpenoid-based insecticides (Ayllón-Gutiérrez et al., 2024).

Although semi-field efficacy was reduced relative to laboratory assays due to environmental degradation of EO constituents (Das and Prakash, 2024), the relative performance gain of the nanoformulation was preserved. Nanoencapsulation not only compensates for this loss but also surpasses free EO in terms of persistence and ultimate mortality. Such enhanced performance is consistent with reports on nanoencapsulated synthetic insecticides (Galloux et al., 2024). The latency phase observed with EO-loaded nanoparticles can be attributed to the slow diffusion properties of biopolymeric carriers, followed by an escalation phase reflecting progressive bioaccumulation and multi-target interactions (Bourenane et al., 2026). Sustained-release technologies, such as EO-based pellets, have similarly been shown to prolong efficacy against pests (Yan et al., 2025).

### 4.5 Implications for IPM

From an IPM perspective, CS–HI nanoparticles represent a promising biorational tool. The use of biodegradable chitosan aligns with sustainable agriculture principles, while sublethal effects (e.g., reduced fecundity) may contribute to pest suppression at lower doses. Although the latency period is longer than synthetic insecticides, it can be strategically managed through earlier application in IPM schedules. Importantly, the CS–HI formulation reduces volatility, improves stability, and enables realistic integration into IPM programs. This outcome aligns with recent updates on sustainable crop protection strategies, which emphasize the importance of environmentally compatible formulations for effective pest management (Aioub et al., 2024; Ayllón-Gutiérrez et al., 2024; Zhou et al., 2024).

Building on these findings, future research should validate efficacy against resistant field populations, assess non-target toxicity on beneficial insects, and evaluate long-term storage stability and compatibility with standard spraying technologies. Importantly, the nanoformulations demonstrated prolonged activity beyond 72 h, underscoring their enhanced stability under semi-field conditions and reinforcing their potential as durable, environmentally compatible alternatives to conventional insecticides (Ayllón-Gutiérrez et al., 2024).

## 5 Conclusion

This study demonstrates that *H. italicum* essential oil nanoencapsulated in chitosan (CS–HI) provides effective control of *A. spiraecola*, achieving complete mortality at 0.5 mg/mL after 140 h exposure and showing clear dose-dependent efficacy at lower concentrations. The formulation’s sustained release profile, reflected in reduced LC_50_ values over time and prolonged activity under semi-field conditions, highlights the reservoir function of chitosan in protecting volatile constituents. Physicochemical characterization confirmed high encapsulation efficiency (97.85%), favorable loading capacity (20.97%), and excellent colloidal stability (zeta potential +45.49 mV, PDI 0.27), supporting both performance and scalability. Compared to unencapsulated oil, CS–HI nanoparticles exhibited enhanced persistence despite a longer latency period, thereby overcoming the volatility limitations of essential oils while maintaining botanical safety (OECD 208 LD_50_ >2000 mg/kg). Collectively, these findings validate nanoencapsulation as a robust strategy for prolonging efficacy and ensuring sustainable citrus protection, establishing a solid foundation for future development of reduced-risk botanical insecticides.

As summarized in **Table 10**, the CS–HI nanoparticles exhibited superior encapsulation efficiency and colloidal stability compared to previous reports. Notably, the lower LD_50_ and higher field efficacy retention highlight the robustness of the formulation under semi−field conditions.

**Table 10:** Comparative analysis of physicochemical and biological parameters between the current study and previous reports.

Parameter	Current study	Previous studies	Reference
Encapsulation efficiency	97.85%	60–90%	^39,60^
Zeta potential	+45.49 mV	+32 to +39 mV	^61,62^
LD_50_ (nanoencapsulated EO)	0.35 mg mL^-^¹ at 96 h	0.5–1.2 mg mL^-^¹	^36,63^
Caryophyllene binding energy	−7.10 kcal mol^-^¹	−6.2 to −6.8 kcal mol^-^¹	^49^
Field efficacy retention	72% (vs. lab)	50–60%	^64,65^

LD_50_ corresponds to the dose that reduce the population 50 %.

## Data Availability

The original contributions presented in the study are included in the article/**Supplementary Material**. Further inquiries can be directed to the corresponding author.

## Glossary

AChE: acetylcholinesterase
ANOVA: analysis of variance
ATR: attenuated total reflectance
CS: chitosan (poly-D-glucosamine)
CS–HI: EO-loaded nanoparticles
CS–NP: chitosan nanoparticle
CTV: Citrus tristeza virus
df: degrees of freedom
DLS: dynamic light scattering
EDX: energy-dispersive X-ray spectroscopy
EE: encapsulation efficiency
EO: essential oil
EO–CS–NP: nanoencapsulated EO
FESEM: field emission scanning electron microscopy
FTIR: Fourier-transform infrared
GABA: neurotransmitter gamma-aminobutyric acid
GC–MS: Gas Chromatography–Mass Spectrometry
LD_50_: letal dose, the dose that kills 50% of the test organisms
LD_90_: letal dose, the dose that kills 90% of the test organisms
NIST: Tukey’s Honest Significant Difference test, National Institute of Standards and Technology
PDB: Protein Data Bank
PL: payload loading
PDBQT format: is a specialized, text-based file format used primarily in molecular docking simulations
PDI: polydispersity index
T80: Tween 80 emulsifier
TPP: tripolyphosphate
UV–Vis spectroscopy: ultraviolet (UV) and visible (Vis) light spectroscopy.
