# Supplementary material for: Nanoencapsulation of Helichrysum italicum (Roth) G. Don essential oil for eco-friendly control of citrus aphid (Aphis spiraecola)
Source: Front Plant Sci. 2026 Apr 13;17:1808571. doi: 10.3389/fpls.2026.1808571 (PMC13111346; doi:10.3389/fpls.2026.1808571)
Supplement: Supplementary file 1 [file DataSheet1.docx]

Supplementary Material

**Green Nanotechnology: Nanoencapsulation of *Helichrysum italicum* Essential Oil for Eco-Friendly Control of Citrus Aphids**


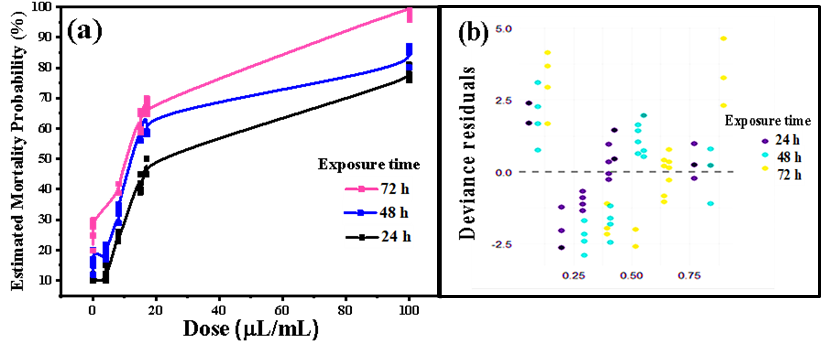


****Figure S1**. Generalized linear model (GLM) analysis describing the dose–time–mortality relationship of *H. italicum* essential oil.**

**Table S1.** Calculated encapsulation efficiency (EE%) and loading capacity (LP%) of CS–HI nanoparticles.

|  | **EL1** | **EL2** | **EL3** | **T** | **Mean** | **Corrected Abs** |
| --- | --- | --- | --- | --- | --- | --- |
| 254 nm | 3.428 | 2.121 | 1.13 | 0.016 | 2.22 | 2.21 |
| 255 nm | 3.189 | 2.012 | 1.072 | 0.016 | 2.091 | 2.075 |
| 256 nm | 3.042 | 1.914 | 1.023 | 0.016 | 1.993 | 1.977 |

EL: The wavelength calculation was repeated three times to ensure accuracy and reproducibility of the results.


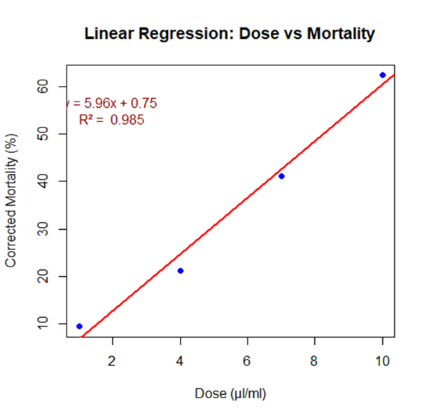

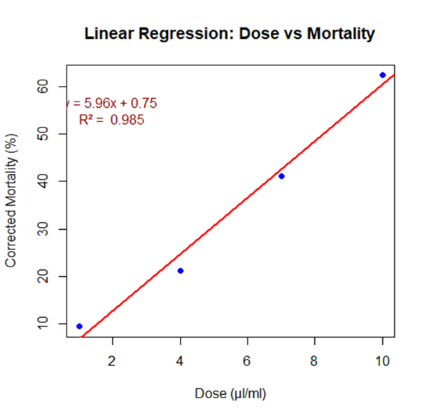


*
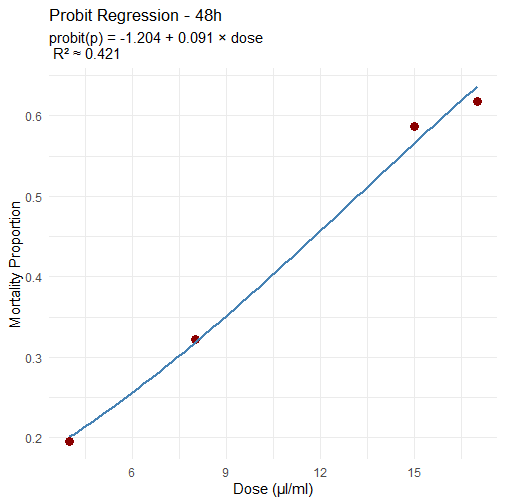
***Figure S2. *In vitro* probit regression curves for *Aphis spiraecola* mortality at 60 and 72 h following treatment with *H. italicum* essential oil (EO).**


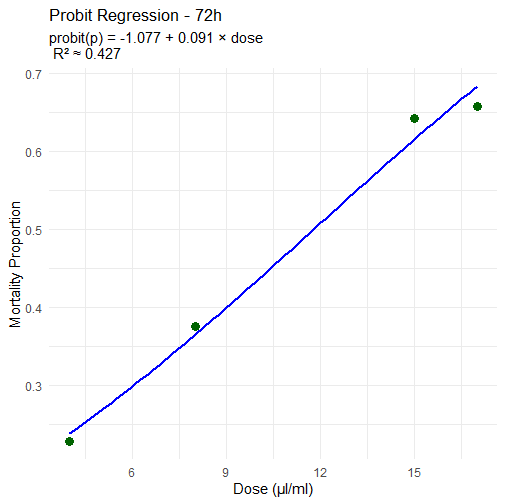


**Figure S3. *In vivo* probit regression curves for *Aphis spiraecola* mortality at 48 and 72 h following treatment with *H. italicum* essential oil (EO).**


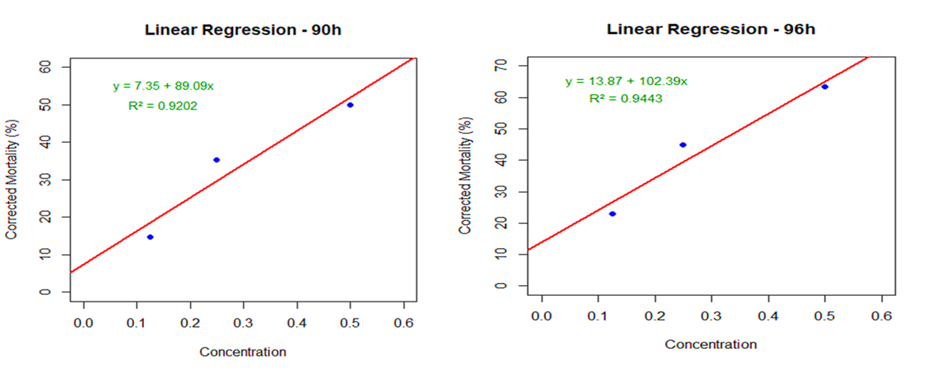


**Figure S4. *In vitro* probit regression curves for *Aphis spiraecola* mortality at 90 and 96 h following treatment with chitosan–encapsulated *H. italicum* essential oil nanoparticles (CS–HI).**
